# Supplementary material for: Effects of Trichoderma harzianum combined with Phanerochaete chrysosporium on lignin degradation and humification during chicken manure and rice husk composting
Source: Front Microbiol. 2025 Feb 28;16:1515931. doi: 10.3389/fmicb.2025.1515931 (PMC11906335; doi:10.3389/fmicb.2025.1515931)
Supplement: Supplementary file 1 [file Data_Sheet_1.zip › 6.Function/All/FunGuild/l2/barplot_sample.pdf]

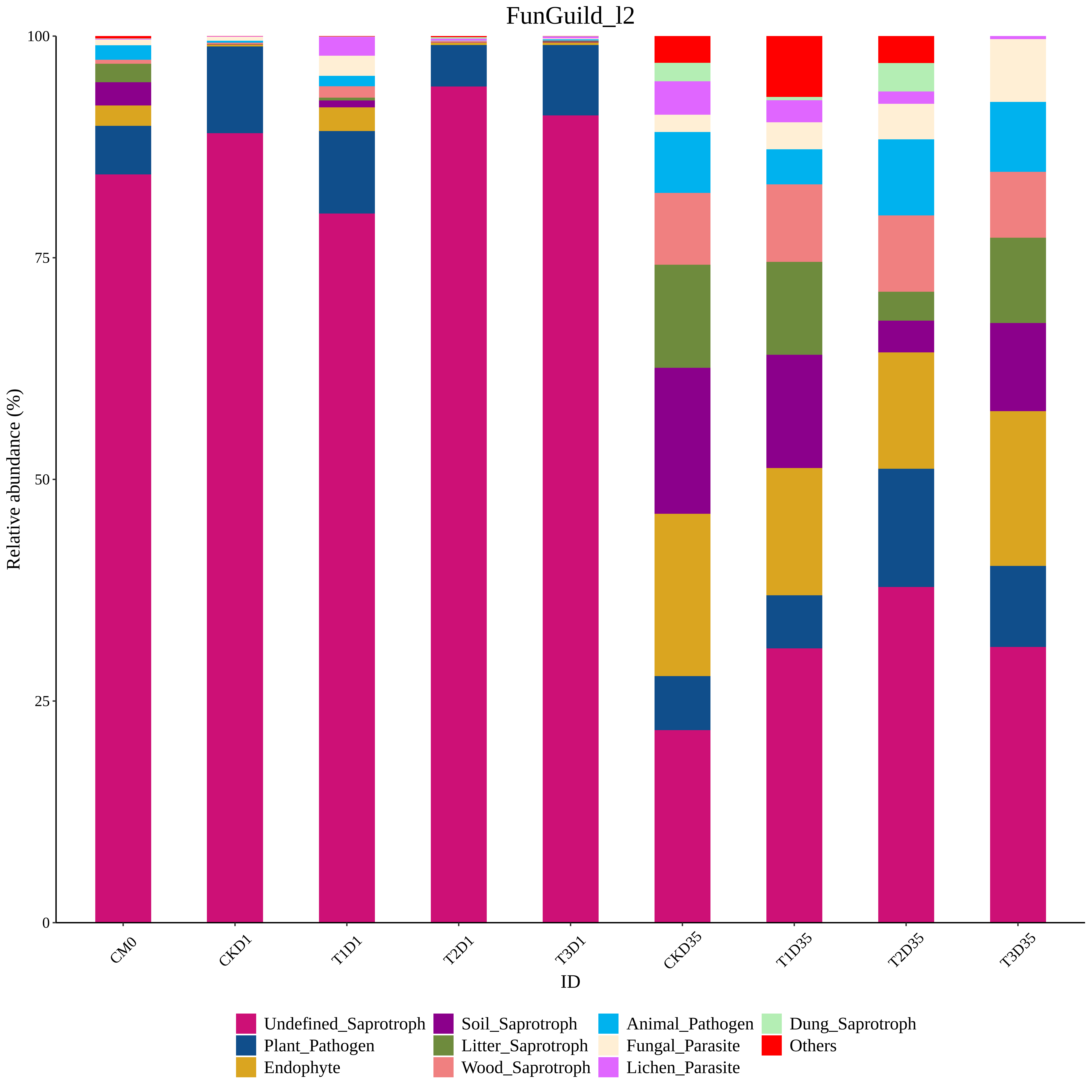
FunGuild_l2
Relative abundance (%)
100
75
50
25
0
CM0
CKD1
T1D1
T2D1
T3D1
CKD35
T1D35
T2D35
T3D35
ID
Undefined_Saprotroph
Plant_Pathogen
Endophyte
Soil_Saprotroph
Litter_Saprotroph
Wood_Saprotroph
Animal_Pathogen
Fungal_Parasite
Lichen_Parasite
Dung_Saprotroph
Others
